# Frequency and Prognostic Significance of Genetic Abnormalities in a Subgroup of Patients With Intermediate-Risk Neuroblastoma: A SIOPEN Study

**DOI:** 10.1200/PO-26-00045

**Published:** 2026-07-01

**Authors:** Hannah E. Hartley, Fang Chyi Fong, Alem S. Gabriel, Louise K. Stevenson, Emily Beckett, Lisa M. Allinson, Angharad Goodman, Aaron Potts, Emily Whittle, Ruby Barford, Alice Lamparelli, Fiona Herd, Katia Mazzocco, Annalisa Pezzolo, Martina Morini, Martina Ardito, Alessandra Eva, Marzia Ognibene, Matthias Fischer, Sandra Ackermann, Carolina Rosswog, Barbara Hero, Graham R. Smith, Adrienne A. Unsworth, Michael McCorkindale, Sally L. George, Jennifer Tall, Jan J. Molenaar, Yvette Matser, Karin Langenberg, Godelieve Tytgat, Rosa Noguera, Ana P. Berbegall, Jaime Font de Mora, Valérie Combaret, Gaelle Pierron, Annick Mühlethaler-Mottet, Jacqueline Schoumans, Joëlle Tchinda, Irina Banzola, Marta Jeison, Michal Hameiri-Grossman, Klaus H. Beiske, Nathalie Auger, Nadine Van Roy, Nermine O. Basta, Richard J. Q. McNally, Sabine Taschner-Mandl, Vassilios Papadakis, Andrea Di Cataldo, Kate Wheeler, Jose D. Bermúdez, Maja Beck-Popovic, Vanessa Segura, Adela Canete, Gudrun Schleiermacher, Deborah A. Tweddle

**Affiliations:** ^1^ Wolfson Childhood Cancer Research Centre, Translational and Clinical Research Institute, Centre for Cancer, Newcastle University, Newcastle Upon Tyne, United Kingdom; ^2^ Children's Cancer Institute, Health Translational Hub, Randwick, NSW, Australia; ^3^ Research Department, National Cancer Council (MAKNA), Kuala Lumpur, Malaysia; ^4^ Newcastle Genetics Laboratory, Newcastle Upon Tyne Hospitals NHS Trust, Newcastle Upon Tyne, United Kingdom; ^5^ Department of Paediatric Oncology, Royal Aberdeen Children's Hospital, Aberdeen, Scotland, United Kingdom; ^6^ Department of Pathology, IRCCS Istituto Giannina Gaslini, Genova, Italy; ^7^ Medical Genetics Unit, IRCCS Istituto Giannina Gaslini, Genova, Italy; ^8^ Laboratory of Experimental Therapies in Oncology, IRCCS Istituto Giannina Gaslini, Genova, Italy; ^9^ Scientific Directorate, IRCCS Istituto Giannina Gaslini, Genova, Italy; ^10^ Department of Experimental Paediatric Oncology, University Children's Hospital, Medical Faculty, University of Cologne, Cologne, Germany; ^11^ Center for Molecular Medicine Cologne (CMMC), Medical Faculty, University of Cologne, Cologne, Germany; ^12^ Department of Pediatric Oncology, University Children's Hospital of Cologne, Medical Faculty, University of Cologne, Cologne, Germany; ^13^ Bioinformatics Support Unit, The Medical School, Newcastle University, Newcastle Upon Tyne, United Kingdom; ^14^ Division of Clinical Studies, The Institute of Cancer Research, London, United Kingdom; ^15^ Children and Young People's Unit, Royal Marsden NHS Foundation Trust, London, United Kingdom; ^16^ Princess Máxima Center for Pediatric Oncology, Utrecht, the Netherlands; ^17^ Department of Pharmaceutical Sciences, Utrecht University, Utrecht, the Netherlands; ^18^ Department of Genetics, Utrecht University Medical Center, Utrecht University, Utrecht, the Netherlands; ^19^ Department of Pathology, Medical School, University of Valencia-Incliva Health Research Institute/CIBERONC, Madrid, Spain; ^20^ Clinical and Translational Research in Cancer, Instituto de Investigación Sanitaria La Fe, Valencia, Spain; ^21^ Laboratoire de Recherche Translationnelle, Centre Léon-Bérard, Lyon, Auvergne-Rhone-Alpes, France; ^22^ Unité de Génétique Somatique, Institut Curie, Paris, France; ^23^ Pediatric Hematology-Oncology Research Laboratory, Woman-Mother-Child Department, Lausanne University Hospital and University of Lausanne, Lausanne, Switzerland; ^24^ Oncogenomics Laboratory, Hematology Service, Department of Laboratory Medicine and Pathology, University Hospital CHUV, Lausanne, Switzerland; ^25^ University Children's Hospital Zurich, Oncology Laboratory, Zurich, Switzerland; ^26^ SPHO Biobank Network, University Children's Hospital Zurich, Oncology Department, Zurich, Switzerland; ^27^ Schneider Children's Medical Center of Israel, Petah Tikva, Israel; ^28^ Department of Pathology, Oslo University Hospital, Medical Faculty, University of Oslo, Oslo, Norway; ^29^ Laboratoire de Cytogénétique, Gustave Roussy, Villejuif, France; ^30^ Center for Medical Genetics, Ghent University, Ghent, Belgium; ^31^ Population Health Sciences Institute, Newcastle University, Newcastle Upon Tyne, United Kingdom; ^32^ St Anna Children's Cancer Research Institute, Vienna, Austria; ^33^ Department of Paediatric Haematology and Oncology (TAO), Marianna V Vardinoyannis-ELPIDA Oncology Hospital, Agia Sofia Children's Hospital, Athens, Greece; ^34^ Department of Clinical and Experimental Medicine, Unit of Paediatric Haematology and Oncology, University Policlinico, Catania, Italy; ^35^ Department of Paediatric Oncology, Oxford Children's Hospital, Oxford, United Kingdom; ^36^ Department of Statistics, University of Valencia, Valencia, Spain; ^37^ Unit of Pediatric Hematology-Oncology, University Hospital CHUV, Lausanne, Switzerland; ^38^ Clinical and Translational Research in Cancer Group, Paediatric Oncology, Instituto de Investigación Sanitaria La Fe, Valencia, Spain; ^39^ Paediatric Oncology Unit, Hospital Universitario y Politecnico La Fe and POG Department, University of Valencia, Valencia, Spain; ^40^ SIREDO Integrated Pediatric Oncology Center and Translational Research in Pediatric Oncology, U1330, INSERM, Institut Curie, Paris, France; ^41^ Great North Children's Hospital, Newcastle Upon Tyne, United Kingdom

## Abstract

**PURPOSE:**

Intermediate-risk neuroblastoma patients older than 18 months, with non–*MYCN* amplified, International Neuroblastoma Risk Group Staging System localized, unresectable or International Neuroblastoma Staging System stage 3 tumors, and unfavorable histology have inferior outcomes compared with other intermediate-risk patients. This study aimed to identify genetic prognostic biomarkers within this rare subgroup.

**METHODS:**

We conducted a large, international study including chromosomal copy number in all cases, next-generation DNA sequencing in most, and telomere maintenance mechanisms and gene expression in a subset, and correlated results with patient survival.

**RESULTS:**

Among 98 tumors, 9/98 (9.2%) had oncogene amplifications (*CDK4/MDM2/TERT* coamplification (n = 1), *CDK4/MDM2* coamplification (n = 4), *CDK4* (n = 2), *TERT* (n = 1), and *MYC* (n = 1)), while 63/98 (64.3%) had typical segmental chromosomal aberrations (tSCAs). Patients with tumors with oncogene amplification had the worst 5-year event-free survival (EFS; 0%; *P* < .0001 log-rank test) and 5-year overall survival (OS; 44.4% [95% CI, 21.4 to 92.3]; *P* < .01 log-rank test). Patients with tumors harboring tSCAs had inferior EFS compared with those with numerical chromosomal aberrations only (51.7% [95% CI, 40.6 to 65.8] *v* 93.3% [95% CI, 81.5 to 100]; *P* < .01). Patients with p53 pathway tumor alterations (n = 10) had worse EFS than those without (0% *v* 61.1% [95% CI, 50.3 to 74.3]; *P* < .0001, log-rank test) and worse OS (26.7% [95% CI, 8.9 to 80.3] *v* 80.9% [95% CI, 71.8 to 91.3]; *P* < .001 log-rank test). Multivariable analysis identified tSCAs as an independent prognostic variable for EFS and oncogene amplification or p53 pathway abnormalities as independent prognostic variables for EFS and OS.

**CONCLUSION:**

Oncogene amplification and/or p53 pathway abnormalities and/or typical SCAs identify patients with intermediate-risk neuroblastoma with inferior outcome for whom intensified or alternative treatments should be considered.

## INTRODUCTION

Neuroblastoma is the most common extracranial solid childhood malignancy.^1^ Copy-number alterations (CNAs) drive clinical and genetic heterogeneity, with patient outcomes ranging from spontaneous regression to fatal progression.^2,3^ Established prognostic variables, including age, disease stage, tumor histology, presence of *MYCN* oncogene amplification (MNA), and segmental chromosomal aberrations (SCAs),^3-6^ guide risk stratification into low-, intermediate-, and high-risk disease.^3^

**CONTEXT**

**Key Objective**
Intermediate-risk neuroblastoma includes a rare subgroup of patients older than 18 months at diagnosis with unresectable, localized tumors with unfavorable histology without *MYCN* amplification. This group is treated differently around the world with some patients receiving upfront myeloablative therapy (MAT) in addition to standard treatment with chemotherapy, surgery, radiotherapy, and 13-cis-retinoic acid, but the overall 5-year patient survival with or without MAT is similar (75%). This study aimed to understand the neuroblastoma biology of this rare patient subgroup.
**Knowledge Generated**
In this large European genetic study of 98 patients, we show that patients whose tumors have typical segmental chromosomal aberrations (tSCA), oncogene amplification, or p53 pathway abnormalities had inferior outcomes on multivariable analyses.
**Relevance**
Patients with neuroblastomas with tSCA, oncogene amplification, or p53 pathway abnormalities should be considered for additional or alternative treatments such as chemoimmunotherapy.

Most children with intermediate-risk neuroblastoma have an overall survival (OS) of 80%-90% with modest treatment.^7-11^ However, a rare subset of patients older than 18 months with intermediate-risk neuroblastoma characterized by localized, non-MNA unresectable disease and International Neuroblastoma Pathology Classification (INPC) unfavorable histology (UH) have an inferior OS of approximately 70%, despite treatment intensification in some studies.^7-11^ Importantly, in most of North America, these patients are classified as high-risk and receive myeloablative therapy (MAT) in addition to standard multimodal treatment, whereas in Europe, treatment typically includes chemotherapy, surgery, radiotherapy, and differentiation therapy with 13-*cis*-retinoic acid.^7,11^

Few studies have reported prognostic biomarkers, although a recent study identified presence of tumoral 11q loss as a poor prognostic marker.^11^ Telomere maintenance mechanisms (TMM) are strong indicators of adverse prognosis across all neuroblastoma risk groups,^12-15^ especially when co-occurring with ALK-Ras-MAPK or p53 pathway mutations.^12^

In this study, we examined the genetic landscape of a large European cohort of intermediate-risk, non-MNA, localized, unresectable (L2) or International Neuroblastoma Staging System (INSS) stage 3 neuroblastoma from patients older than 18 months at diagnosis with INPC UH, to identify biological features predicting worse outcome.

## METHODS

### Patients and Tumors

Ninety-eight tumors were included from patients older than 18 months with localized, unresectable International Neuroblastoma Risk Group Staging System L2 or INSS stage 3, non-MNA intermediate-risk neuroblastoma with INPC poorly differentiated or undifferentiated histology diagnosed between 2007 and 2021.

Eighty patients were treated according to international SIOPEN protocols (ClinicalTrials.gov identifier: NCT01728155; n *=* 53) or the UK protocol for low- and intermediate-risk neuroblastoma (n *=* 27; Data Supplement, Table S1).^7,16^ Ten children were enrolled in German Pediatric Oncology and Haematology (GPOH) clinical trials NB2004/NCT00526318 (n *=* 9)^17^ and NB97/NCT00017225 (n *=* 1; Data Supplement, Table S1).^18^ Eight patients were enrolled in clinical trials in the Netherlands and treated according to the Dutch Childhood Oncology Group (DCOG) NBL2009 protocol^19^ (Data Supplement, Table S1). All trials were conducted in accordance with the Declaration of Helsinki and Good Clinical Practice. Tumor and blood samples were obtained from national biobanks (Data Supplement, Supplementary Methods). Most tumor analyses were undertaken within Newcastle University and NHS genetics laboratories (n = 80), with 18 analyzed elsewhere (Germany and the Netherlands).

### Copy-Number (CN) Data

**FIG 1.**
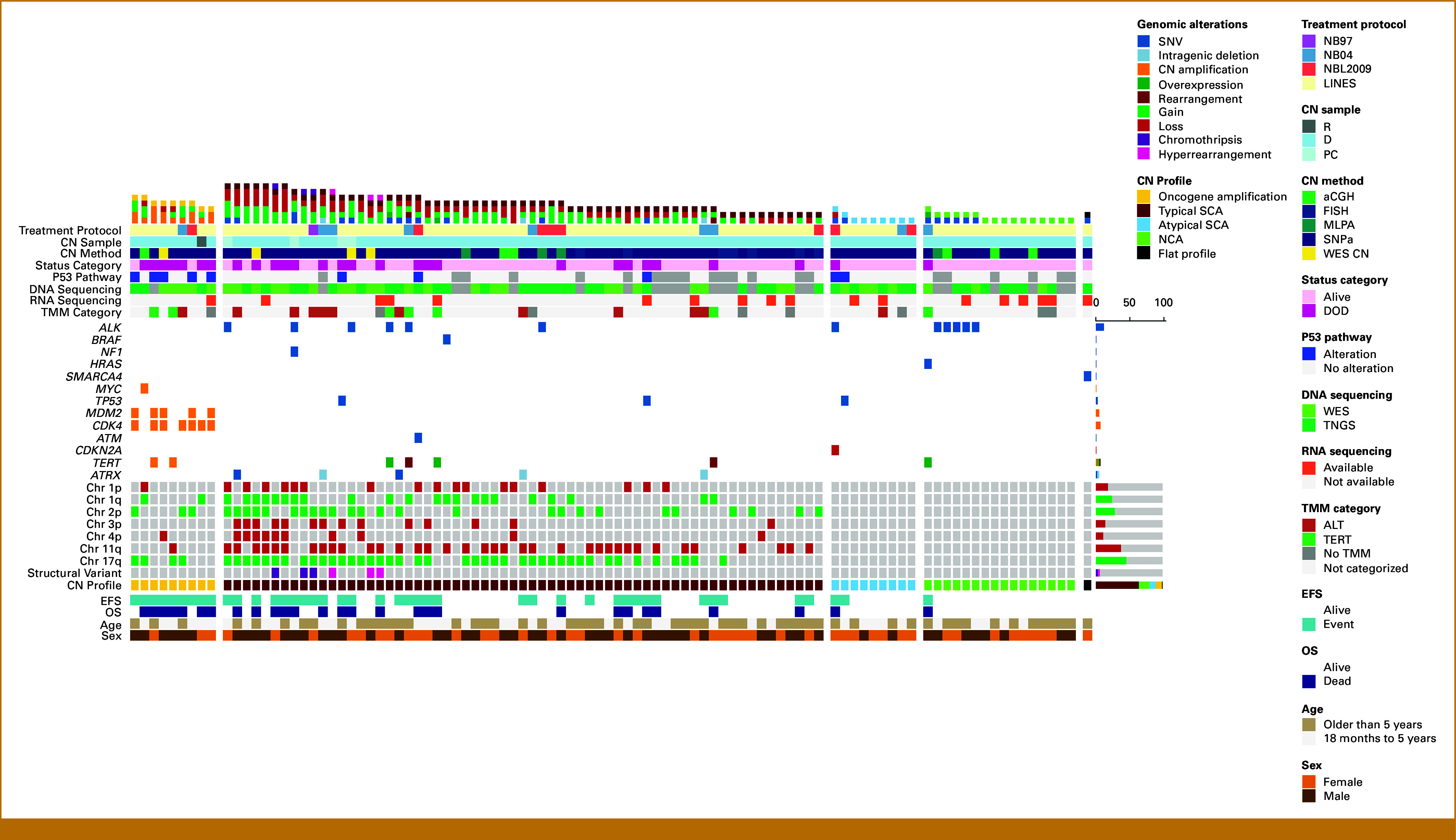
Overview of cohort clinical and genetic profile. Profile heatmap for the whole cohort (N = 98). Chromosomal copy-number alterations are shown in the top panels and the final CN profile categorizations are given below. Somatic *ALK*, RAS/MAPK, and p53 pathway-related genetic alterations are shown in the third panel, followed by TMM categories and associated genetic alterations. The bottom panels show clinical characteristics including EFS, OS, age, and sex. ALT, alternative lengthening of telomeres; CN, copy number; D, diagnosis; DOD, died of disease; EFS, event-free survival; NCA, numerical chromosomal aberration; OS, overall survival; PC, post-chemotherapy; R, relapse; SCA, segmental chromosomal abberation; SNV, single-nucleotide variant; TMM, telomere maintenance mechanisms; TNGS, targeted next-generation sequencing; WES, whole-exome sequencing.

Tumor DNA (Data Supplement, Table S9) was available for CN analysis from all patients at diagnosis (n *=* 93), after chemotherapy (n *=* 4), or relapse in the absence of chemotherapy treatment (n *=* 1). CN analysis (n *=* 98) was undertaken using a combination of SNPa (n *=* 77), array comparative genomic hybridization (aCGH; n *=* 6), multiplex ligation-dependent probe amplification (MLPA; n *=* 4), fluorescence *in situ* hybridization (FISH; n *=* 7), or whole-exome sequencing (WES) to CN (n *=* 4; Fig 1; Data Supplement, Tables S2A and S9, and Fig S1). CN calls and SCA nomenclature are detailed in the Data Supplement (Supplementary Methods). Frequently occurring SCAs in neuroblastoma, classified as typical (tSCAs) by SIOPEN Biology guidelines, were –1p, +1q, +2p, –3p, –4p, –11q, and +17q.^4,5^ Any other SCAs were defined as atypical SCAs.

### DNA Sequencing

Seventy-eight tumors had DNA sequencing undertaken, WES on both tumor and germline DNA (n *=* 27), or tumor DNA only if germline DNA was unavailable (n *=* 3). Targeted NGS (TNGS) was undertaken on tumor DNA (n *=* 31) using a panel of 132 genes (Data Supplement, Table S10A). Further details including variant annotation and classification are described in the Data Supplement (Supplementary Methods). Pathogenic or likely pathogenic SNVs affecting *ALK*/Ras-MAPK (11 genes: *ALK, CDK4, FGFR1, NF1, PTPN11, CCND1, LIN28B, BRAF, HRAS, KRAS,* and *NRAS*) or p53 pathways (six genes: *TP53, MDM2, MDM4, ATM, CDKN2A,* and *CREBBP*) were identified. Methylation arrays were undertaken on seven tumors (Data Supplement, Methods).

RNA sequencing was undertaken on 18 tumors. Further details, use of STAR-Fusion and gene expression signatures is described in the Data Supplement (Supplementary Methods).

### TMM Assays and Subgrouping

Tumors (n *=* 27; 28%) were categorized for telomerase activation or alternative lengthening of telomeres (ALT). Fluorescence in situ hybridization for *TERT* rearrangement (*TERT*-FISH), ALT immunoFISH, and C-circle assays are described in the Data Supplement (Supplementary Methods). ALT was assessed using ALT immuno-FISH (n *=* 24) and C-circle qPCR (n *=* 3; Data Supplement, Fig S1). Tumors with either *ATRX* pathogenic mutations or intragenic deletions, and/or ALT immuno-FISH positive score, were defined as ALT-positive. *TERT-*positive tumors comprised those with *TERT* rearrangement by *TERT*-FISH, *TERT* amplification by SNPa, or high *TERT* mRNA expression (Data Supplement, Fig S5D). Samples testing negative for *TERT*-FISH, lacked *TERT* amplification or high *TERT* expression, and scored negative for ALT immuno-FISH were classified as TMM-negative (n *=* 8).

### Statistics

Event-free survival (EFS) was defined as the time from diagnosis to first event (progression or relapse or second malignancy) or last follow-up. OS was defined as the time from diagnosis to death or last follow-up (31/1/2024). OS and EFS were analyzed according to the Kaplan-Meier method and compared using the log-rank test for significance. *P* values for all univariate survival analyses with two-arm comparisons were pooled and adjusted using the Benjamini-Hochberg method for false discovery rate (Data Supplement, Table 12). To analyze the impact of biological and clinical factors, multivariable Cox regression analysis was carried out using statistically significant variables from univariate survival analysis, excluding TMM classification and gene expression classifiers due to low numbers (n = 27 and n = 18, respectively). Contingency tables were analyzed using the *X*^2^ or Fisher's exact test. All statistical analyses were conducted in R (v4.0.3), with results considered statistically significant when adjusted, if *P* < .05.

### Ethics Approval and Patient Consent

This study was approved by Newcastle University Ethics Committee (7579/2020) and all biobanks listed above that had integrated ethics approval (23/EM/0130 for VIVO biobank). All patients and families consented to inclusion of their tumors in the study either through the ethically approved biobank or other studies.

## RESULTS

### Patient Cohort

Ninety-eight patients with intermediate-risk neuroblastoma with a median age at diagnosis of 47.3 months (range, 18.1-204.5 months) were included. Among survivors, the median follow-up time was 82.3 months (range, 10.6-200.9 months; Table 1). Five-year EFS and OS of the whole cohort was 56.4% (95% CI, 47.3 to 67.2) and 76.3% (95% CI, 68.0 to 85.5), respectively. Relapse occurred in 42/98 (43.9%) patients, most commonly local (24/42; 57.1%), followed by metastatic (10/42; 23.8%) and combined (6/42; 16.7%) relapses (Table 1; Data Supplement, Table S1).

**TABLE 1.** Clinical Characteristics by Treatment Protocol

Characteristic	SIOPEN (including UK cases; n = 80)	NB-2004 (n = 9)/NB-97 (n = 1)/NBL-2009 (n = 8)	Whole Cohort (N = 98)
Sex			
Male	42	10	52
Female	38	8	46
Age at diagnosis			
18 months-5 years	58	7	65
>5 years	22	11	33
Relapse			
Yes	30	12	42
No	50	6	56
Type of relapse			
Combined	6	1	8
Metastatic	8	2	10
Local	15	9	24

Abbreviations: NB2004/NB-97, German Paediatric Oncology protocol; NBL-2009, Dutch Paediatric Oncology Protocol; SIOPEN, International Society of Pediatric Oncology Group.

### CN Abnormalities

**FIG 2.**
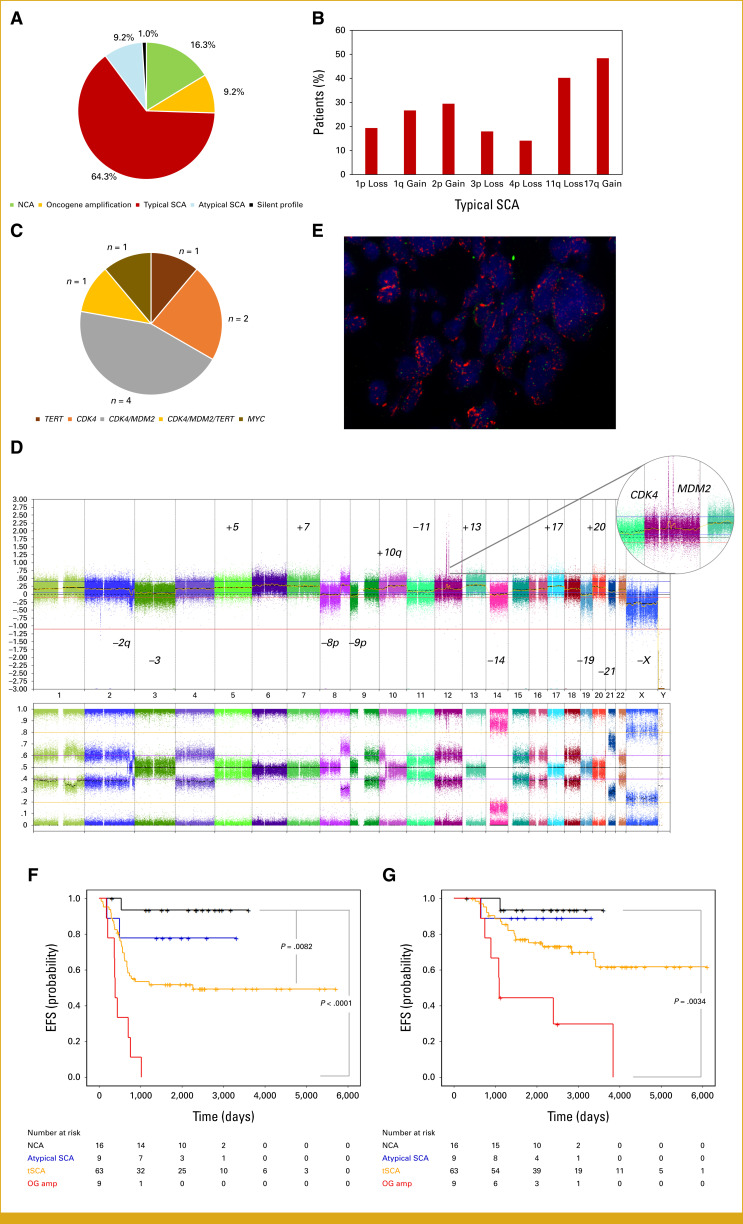
(A) Copy-number profile categories within the whole cohort (N = 98). (B) Frequencies of typical SCAs. (C) Oncogene amplifications include *CDK4/MDM2/TERT* (n = 1), *CDK4/MDM2* (n = 4), *CDK4* (n = 2), *TERT* (n = 1), and *MYC* (n = 1) amplification. (D) SNPa for a case with *CDK4/MDM2* coamplification on 12q14.1 and 12q15 loci, respectively. The top panel shows the log2 ratio for probe signal intensity against the intensity at the diploid baseline, indicating changes in copy number. The bottom panel shows the B-allele frequencies. Amplification is defined using a log2 ratio of ≥0.7. (E) FISH image showing *CDK4* amplification. Red probe represents *CDK4*; green probe represents chr12 centromere. Kaplan-Meier survival curve comparing (F) EFS and (G) OS between copy-number profile subgroups. *P* values were calculated via log-rank test corrected for multiple testing (pairwise comparison, Benjamini-Hochberg correction). EFS, event-free survival; FISH, fluorescence in situ hybridization; NCA, numerical chromosomal aberrations; OG amp, oncogene amplification; OS, overall survival; SCA, segmental chromosomal aberrations; SNPa, single-nucleotide polymorphism array; tSCA, typical segmental chromosomal aberrations.

We analyzed the CN profiles of all 98 tumors (Fig 1; Data Supplement, Fig S1, Table S2A and S2B). Tumors were categorized as follows: oncogene amplification (9/98; 9.2%), typical SCA (tSCA; 63/98; 64.3%), numerical chromosomal aberrations (NCA) only (16/98; 16.3%), atypical SCA (9/98; 9.2%), or silent genomic profile (1/98; 1.0%; Figs 1, 2A-2C; Data Supplement, Table S2A and S2B). The most frequent tSCAs were 17q gain (45/93; 48.4%), 11q loss (37/92; 40.2%), 2p gain (28/95; 29.5%), 1q gain (24/90; 26.7%), 1p loss (18/93; 19.4%), 3p loss (14/88; 15.9%), and 4p loss (11/88; 12.5%; Figs 1, 2A-2C). Of tumors with an NCA-only profile, gains of whole chromosome 17 (13/16; 81.3%), chromosome 7 (11/16; 68.8%), and chromosome 18 (10/16; 62.5%) were most frequent (Data Supplement, Fig S2A). The most frequent atypical SCA was 19p loss (Data Supplement, Fig S2A).

Patients whose tumors harbored 11q loss had inferior EFS, with a 5-year EFS of 38.8% (95% CI, 25.7 to 58.7) versus 66.8% (95% CI, 55.4 to 80.6) for those without 11q loss (*P* = .049, log-rank test). Patients whose tumors had 17q gain had a 5-year EFS of 40.8% (95% CI, 28.5 to 58.4) versus 70.3% (95% CI, 58.4 to 84.7) for those without 17q gain (*P* = .045, log-rank test). There was no difference in OS for patients with tumors with either 11q loss of 17q gain v those without (Data Supplement, Figs S2C and S2D, and Table S3). Patients with tumors with a segmental profile, defined as any SCAs with or without NCAs, compared with a nonsegmental profile, were significantly associated with inferior 5-year EFS, 48.8% (95% CI, 39.0 to 61.1) versus 93.8% ([95% CI, 82.6 to 100.0]; *P* = .01, log-rank test), but not OS (Data Supplement, Figs S2D, S2E and Table S3). One tumor (NB85) exhibited a silent genomic profile without detectable NCAs or SCAs despite 90% tumor cell content (Data Supplement, Fig S3A). TNGS identified a nonsense mutation in *SMARCA4* (p.R1618Ter; c.4852C>T) with a variant allele frequency (VAF) of 90.6% (Data Supplement, Table S4) associated with a 1.45-Mb copy-number loss at 19p13.2 encompassing *SMARCA4* (Data Supplement, Figs S3A-S3C), resulting in biallelic inactivation. *SMARCA4* expression was reduced relative to other tumors, consistent with nonsense-mediated decay (Data Supplement, Table S5 and Fig S3D).

Chromothripsis, a complex structural event involving tens to thousands of genomic rearrangements,^14,32^ was identified in 3/87 (3.4%) tumors involving chromosomes 5, 12, and 19 (Data Supplement, Table S2A). Chromosomal hyper-rearrangement was identified in four additional tumors, including one with oncogene amplification.

#### Oncogene Amplification Identifies a Subgroup With Inferior Outcome

Amplification of known neuroblastoma oncogenes was present in 9/98 (9.2%) tumors (Figs 1, 2A-2G; Data Supplement, Table S6A). Recurrent *CDK4*/*MDM2* coamplification at chromosome loci 12q14.1 and 12q15, respectively, was identified in 4/9 (44.4%) oncogene-amplified tumors (Figs 2C-2E; Data Supplement, Table S6A). *CDK4*/*MDM2/TERT* coamplification was identified in one tumor, while *CDK4* amplification alone was present in two tumors. In one *CDK4/MDM2* coamplified tumor for which RNAseq data were available, both *CDK4* and *MDM2* expression were markedly increased compared with other tumors (Data Supplement, Table S5). *TERT* (n *=* 1) and *MYC* (n *=* 1) amplifications were identified at 5p15.33 and 8q24.21 loci, respectively (Figs 1, 2A, 2C; Data Supplement, Table S6A). All oncogene-amplified cases harbored SCAs, most commonly 17q gain (4/9), 2p gain (3/9), and 9p loss (3/9; Data Supplement, Table S2A and S2B). Pairwise log-rank comparisons were conducted between CN profiles (n *=* 97; Data Supplement, Table S6B). Patients whose tumors had tSCA had a 5-year EFS of 51.7% (95% CI, 40.6 to 65.8) and 5-year OS of 75.1% (95% CI, 65.0 to 86.9). Patients whose tumors had atypical SCA had a 5-year EFS of 77.8% (95% CI, 54.9 to 100.0) and 5-year OS of 88.9% (95% CI, 70.6 to 100.0; Data Supplement, Table S3). No significant EFS and OS differences were found between patients whose tumors had atypical SCA and tSCA profiles (Figs 2F-2G; Data Supplement, Tables S3 and S6B).

Strikingly, patients whose tumors had oncogene amplifications had a 5-year EFS of 0% and 5-year OS of 44.4% (95% CI, 21.4 to 92.3), and the worst EFS (*P* < .0001, log-rank test) and OS (*P* = .003, log-rank test; Figs 2F and 2G; Data Supplement, Table S6B).Patients with tumors with NCA only had a 5-year EFS of 93.3% (95% CI, 81.5 to 100) and 5-year OS of 93.3% (95% CI, 81.5 to 100.0; Figs 2F and 2G; Data Supplement, Table S3B), and significantly better EFS than patients with tSCAs (*P* = .008, log-rank test; Fig 2F; Data Supplement, Table S6B).

#### p53 Pathway Abnormalities But Not *ALK* Mutations Identify a Poor Prognostic Group

**FIG 3.**
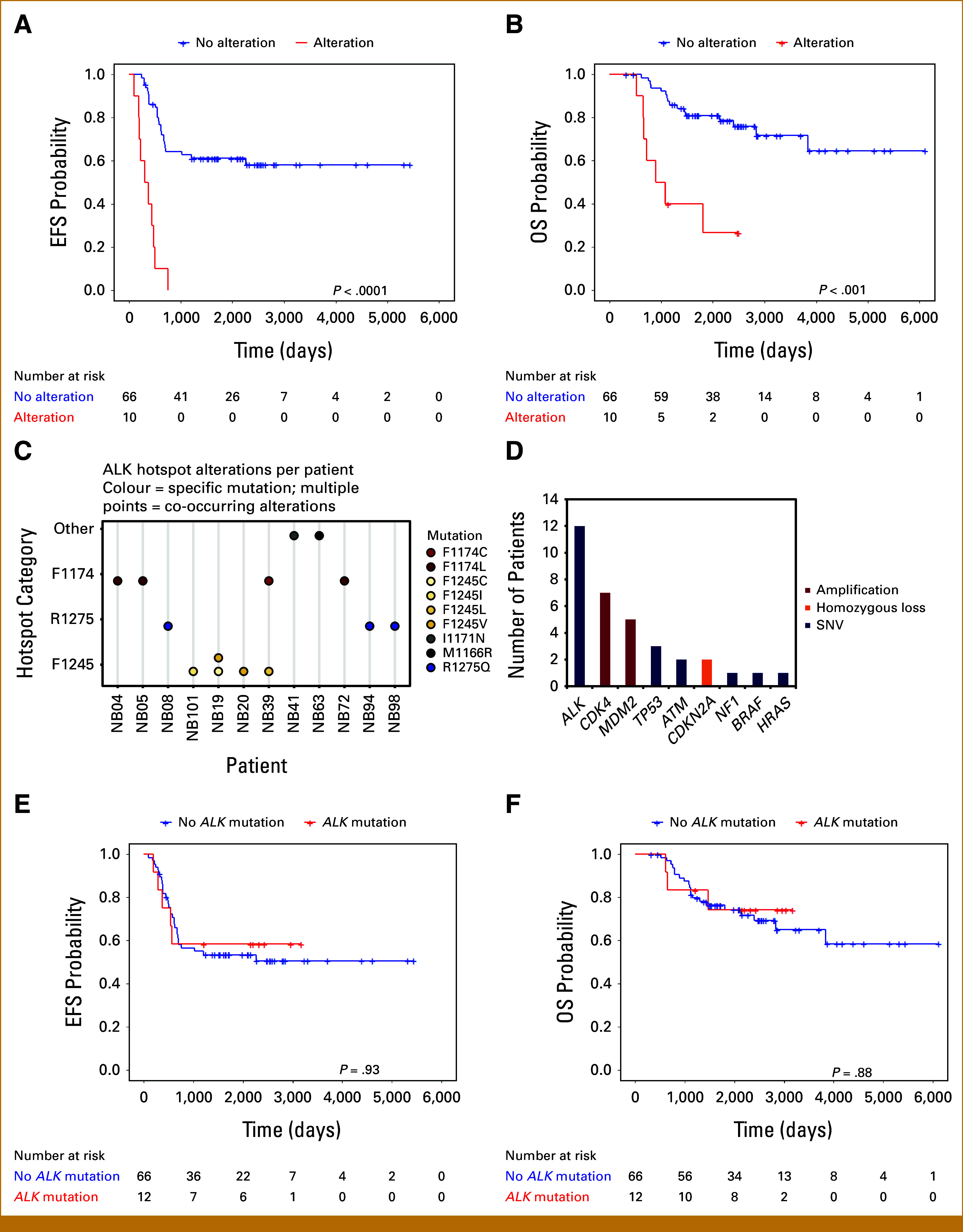
Alterations in p53 and RAS-MAPK pathway. Kaplan-Meier survival curves comparing (A) EFS and (B) OS of patients with tumors with or without somatic p53 pathway alterations (n = 76; log-rank test). p53 pathway alterations include *CDK4/MDM2* coamplification (n = 5), *TP53* mutations (n = 3), *CDKN2A* homozygous loss (n = 1), and *ATM* (n = 1). (C) Breakdown of *ALK* mutation types and their frequencies. R1275Q (n = 3), F1174C (n = 1), F1174L (n = 3), F1245V (n = 2), F1245C (n = 1), F1245I (n = 1), and F1245L (n = 1). One case, NB39, harbored two *ALK* mutations, F1245L (VAF = 27.2%) and F1174C (VAF = 5.7%). In NB19, an F1245C mutation was identified by Sanger sequencing, while Agena MassARRAY analysis revealed base substitutions at positions c.3733T>G (VAF = 11%) and c.3734T>G (VAF = 26%). (D) Frequencies of *ALK/RAS-MAPK* and p53 alterations. An *NF1* mutation (R2258Ter nonsense mutation) co-occurred with an *ALK* mutation (R1275Q) in one case, while *CDKN2A* biallelic loss co-occurred with an *ALK* mutation (F1245I) in another. (E) EFS and (F) OS in patients according to the presence of an activating *ALK* mutation (n = 78; log-rank test). EFS, event-free survival; OS, overall survival; VAF, variant allele frequency.

Patients with tumors with p53 pathway abnormalities defined as *TP53* inactivating mutations, *MDM2* amplification, homozygous *CDKN2A* deletion, or pathogenic *ATM* mutations (n = 10) had inferior 5-year EFS and OS compared with patients with tumors without p53 pathway abnormalities (0% *v* 61.1% [95% CI, 50 to 74.3]; *P* < .0001, and 26.7% [95% CI, 8.9 to 80.3] *v* 80.9% [95% CI, 71.8 to 91.3]; *P* = .0003, respectively n *=* 76; Figs 3A and 3B; Data Supplement, Table S3). After excluding five cases with oncogene amplification (n *=* 67), p53 pathway alterations remained significantly associated with inferior EFS and OS (Data Supplement, Fig S4A and Table S3). Three tumors showed pathogenic loss-of-function *TP53* mutations, including missense mutations, P152L (VAF = 17.9%) and C141Y (VAF = 98.3%), both of which co-occurred with whole chromosome 17 LOH, as well as one tumor with C135Y (VAF = 59.8%; Data Supplement, Table S4). Low VAF for *TP53* p.P152L may reflect a subclonal variant.

Gain-of-function *ALK* mutations within the tyrosine kinase domain (TKD) represent the most common acquired single-gene alterations in neuroblastoma.^22^ Among 80 tumors, 12 (15%) carried at least one TKD mutation (Fig 3C; Data Supplement, Table S4). As expected, we did not observe *ALK* amplification in this non-MNA cohort. *ALK* expression was determined for samples with RNA-seq data available (n *=* 18; Data Supplement, Table S5 and Fig S4B). The highest *ALK* expression was identified in the tumor with an I1171N variant, whereas lower expression was identified in NB63 with a M1166R variant (Data Supplement, Table S5 and Fig S4B). In contrast to high-risk neuroblastoma,^12,34^ the presence of a somatic *ALK* mutation alone was not associated with EFS ( 58.3% [95% CI, 36.2 to 94.1] *v* 53.4% [95% CI, 42.5 to 67.1]; *P* = .93, log-rank test) or OS (74.1% [95% CI, 52.6 to 100.0] *v* 74.2% [95% CI, 64.0 to 86.1]; *P* = .88) for patients with and without *ALK* mutations, respectively (Figs 3E and 3F; Data Supplement, Table S3). Pathogenic Ras-MAPK pathway mutations included *BRAF, HRAS*, and *NF1* (Fig 3D; Data Supplement, Table S4).

#### TMM

**FIG 4.**
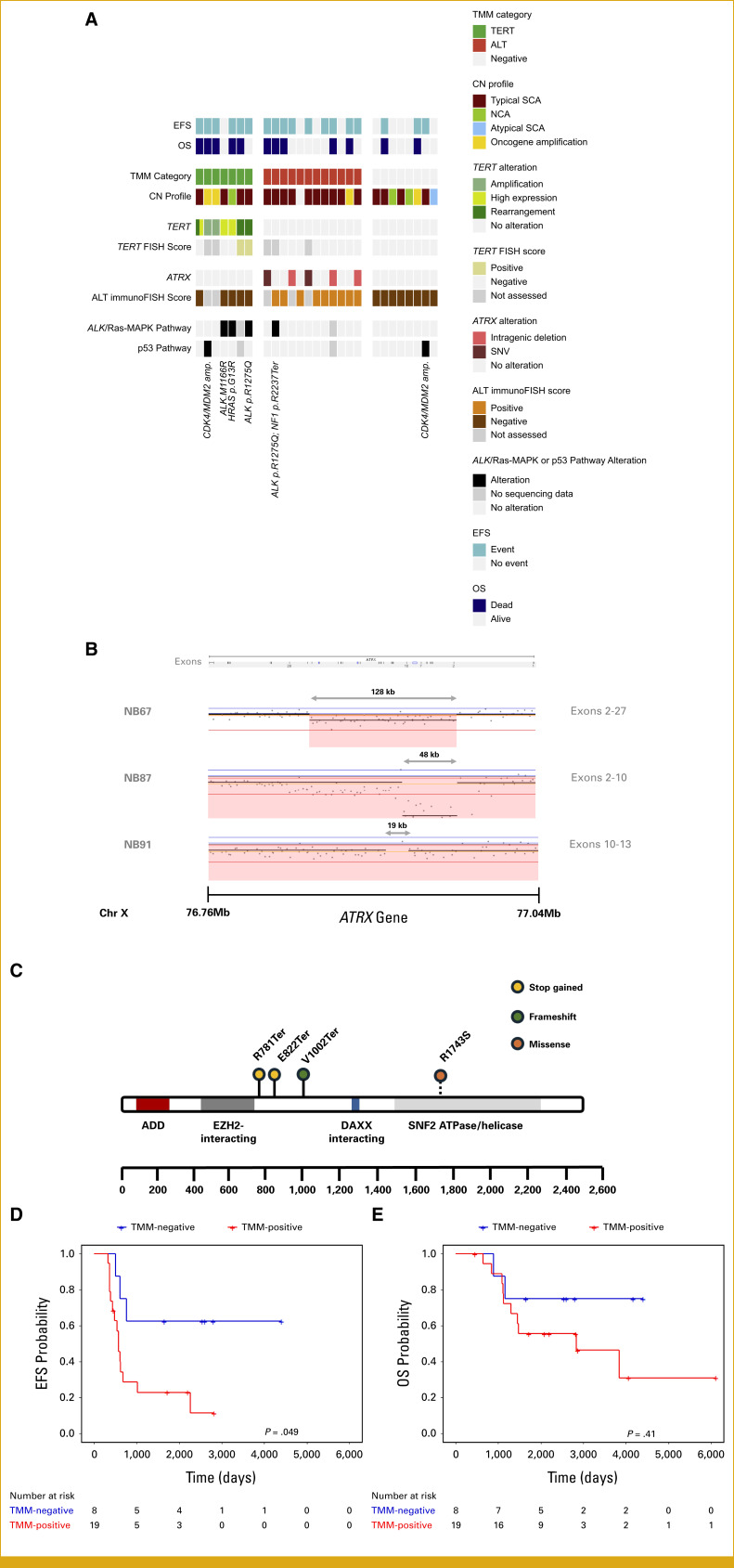
Assessment of TMM in intermediate-risk neuroblastoma (n = 27). (A) Telomere maintenance mechanism overview. Pathogenic or likely pathogenic SNVs or amplifications affecting *ALK*/Ras-MAPK (*ALK, CDK4, FGFR1, NF1, PTPN11, CCND1, LIN28B, BRAF, HRAS, KRAS,* and *NRAS*) or p53 pathways (*TP53, MDM2, MDM4, ATM, CDKN2A,* and *CREBBP*) were assessed. p53 pathway abnormalities identified within this subgroup (n = 27) include *CDK4/MDM2* (n = 1) and *CDK4/MDM2/TERT* (n = 1) coamplification. *ALK*/Ras-MAPK alterations include *ALK* (n = 3), *NF1* (n = 1), and *HRAS* (n = 1): (B) Cases with *ATRX* intragenic deletions. (C) *ATRX* mutations. R1743S is a missense mutation found in NB79, with uncertain pathogenicity, given its absence from COSMIC and ClinVar (tier 3a), and was therefore excluded from the *ATRX*-altered group. A somatic frameshift *ATRX* mutation, V1002Ter (VAF = 20.6%), and a stop-gain E822Ter (VAF = 66.4%) mutation were identified by WES in one tumor. A stop-gain mutation, R781Ter (VAF = 73.3%), was identified by TNGS in another tumor. Kaplan-Meier curves comparing (D) EFS and (E) OS in patients with tumors with or without TMM (n = 27). ADD, ATRX-DNMT3-DNMT3L domain; ALT, alternative lengthening of telomeres; CN, copy number; EFS, event-free survival; OS, overall survival; *TERT*-FISH, fluorescence in situ hybridization for *TERT* rearrangement; TMM, telomere maintenance mechanisms; VAF, variant allele frequency.

19/27 (70.4%) tumors were deemed TMM-positive based on the presence of ALT or telomerase activation (Fig 4A; Data Supplement, Table S7 and Fig S1).

#### Telomerase Activation

Telomerase activation was present in 7/27 (25.9%) tumors through *TERT* rearrangement (n *=* 3), *TERT* amplification (n *=* 2), or high *TERT* expression without detectable *TERT* structural alterations (n *=* 2; Fig 4A). *TERT* rearrangements were identified by a positive *TERT*-FISH score (n *=* 2; Data Supplement, Table S7). There were no *TERT* promoter mutations or *TERT* pathogenic mutations (n *=* 24; Data Supplement, Table S7). One tumor had CN loss at the 5p15.33 to 5p15.1 locus upstream of *TERT*, confirmed by EPIC methylation array CN analysis (Data Supplement, Figs S5A-S5C). This tumor demonstrated high *TERT* expression, but *TERT*-FISH failed to confirm a rearrangement (Data Supplement, Tables S5, S7 and Fig S5D). Instead, we identified a novel intrachromosomal *MARCHF11::CLPTM1L* fusion transcript, suggesting breakpoints upstream of *TERT* at the genomic coordinates 1.34 Mb and 16.18 Mb, matching SNPa CN findings (Data Supplement, Table S8 and Fig S5E). This fusion conforms to known patterns of *TERT*-rearranged neuroblastoma^24^ (Data Supplement, Fig S5E). *MYCN* amplification, another mechanism of telomere maintenance,^25^ prompted investigation of a *MYCN* gene expression signature^48^ (n *=* 18; Data Supplement, Table S5, and Figs S6A and S6B). Tumors with EPIC methylation array data available (n *=* 7) were studied using the Heidelberg classifier.^35^ NB85, classifying as *MYCN*-type, displayed the highest *MYCN* expression and a positive *MYCN* signature,^48^ despite presenting with a silent genomic profile and two *MYCN* FISH signals (Data Supplement, Figs S6A, S6B and Table S5), supporting the presence of significant *MYCN* activity in some non-MNA neuroblastomas.

#### ALT

In our cohort, 12/27 (44.4%) tumors were ALT-positive based on a positive ALT immuno-FISH score (n *=* 7), identification of *ATRX* alterations (n *=* 3), or both (n *=* 2; Figs 4A-4C). Somatic *ATRX* alterations*,* commonly intragenic deletions and small indels, are associated with ALT in neuroblastoma.^26,28^ We identified typical intragenic *ATRX* deletions in exons 2-10, and 10-13,^28^ and atypical patterns in exons 2-27, and *ATRX* mutations (Figs 4B and 4C; Data Supplement, Table S7). Patients with *ATRX* alterations had adverse 5-year EFS compared with those without *ATRX* alterations, 0% versus 56.4% ([95% CI, 45.6 to 69.7]; *P* = .03, log-rank test), but no difference in OS (Data Supplement, Fig S7 and Table S3). ALT was detected in the absence of *ATRX* alteration in 7/12 (58.3%) tumors, based on a positive ALT immuno-FISH score (Fig 4A). We further validated the ALT status of three TMM-categorized tumors using a novel qPCR assay for quantification of ALT-associated C-circles (Data Supplement, Table S7).

Patients with TMM-positive neuroblastoma showed significantly worse 5-year EFS than those without TMM (22.8% [95% CI, 9.7 to 53.7] *v* 62.5% [95% CI, 36.5 to 100.0]; *P* = .049, log-rank test), but not 5-year OS (55.6% [95% CI, 36.8 to 84.0] *v* 75.0% [95% CI, 50.3 to 100.0]; *P* = .41, log-rank test; Figs 4D and 4E; Data Supplement, Tables S3 and S7).

#### Mesenchymal and Adrenergic Signatures

Neuroblastoma cells exhibit plasticity between an adrenergic and a more undifferentiated mesenchymal-like state.^11,36^ RNAseq data were used to distinguish the two differentiation states using previously established mesenchymal and adrenergic gene expression signatures.^36^ We identified five samples with a predominantly mesenchymal expression pattern, while the remainder showed an adrenergic expression pattern (n *=* 13; Data Supplement, Table S5 and Fig S8A). Patients with tumors with a mesenchymal signature had significantly worse 5-year EFS (60.0% [95% CI, 29.3 to 100.0] *v* 91.7% [95% CI, 77.3 to 100.0]; *P* = .042, log-rank test) but not 5-year OS (60.0% [95% CI, 29.3 to 100.0] *v* 91.7% [95% CI, 77.3 to 100.0]; *P* = .24, log-rank test) compared with those with an adrenergic signature (Data Supplement, Fig S8B). Other gene expression signatures have been used for neuroblastoma risk stratification.^37,38^ Based on these signatures, we conducted a principal component (PC) analysis to investigate patient samples according to survival. Clustering was observed in the group who died of disease within a negative PC1/PC2 range, in line with a high-risk stratification^38^ (Data Supplement, Figs S9A and S9B).

#### Multivariable Analyses

Significant variables from both univariate and pairwise survival analyses were included in a multivariable Cox proportional hazards model, but not TMM or cell-state signatures, due to small numbers (Table 2; Data Supplement, Tables S3 and S6B). Oncogene amplification or p53 pathway abnormalities were combined due to overlapping cases. p53 pathway alterations or oncogene amplification (hazard ratio [HR],: 5.77; *P* < .0001) and tSCAs (HR, 3.64; *P* = .008) were prognostic for EFS, but not age >5 years (HR, 1.94; *P* = .06; Table 2). p53 pathway alterations and oncogene amplification were the only independent adverse prognostic markers for OS (HR, 7.00; *P* < .0001; Table 2).

**TABLE 2.** Multivariable Analyses Show the Presence of Either Oncogene Amplification or p53 Pathway Alterations Are Independent Predictors of Inferior Outcome (n *=* 73)

Clinical or Genetic Characteristic	HR	Lower CI (95%)	Upper CI (95%)	*P*
EFS				
Presence of oncogene amplification or p53 pathway alterations	5.77	2.79	11.92	<.0001
tSCA	3.64	1.39	9.53	.008
>5 years old	1.94	0.96	3.91	.06
OS				
Presence of oncogene amplification or p53 pathway alterations	7.01	2.91	16.89	<.0001
tSCA	1.35	0.44	4.17	.6
>5 years old	0.94	0.38	2.31	.9

NOTE. The Cox multiple regression model using a log-rank test score was applied.

Abbreviations: EFS, event-free survival; HR, hazard ratio; OS, overall survival; tSCA, typical segmental chromosomal aberrations.

## DISCUSSION

We conducted a comprehensive genetic study of a large international European cohort of 98 patients with intermediate-risk neuroblastoma older than 18 months at diagnosis, with L2, UH, non-MNA disease who were treated using similar protocols without upfront MAT. The aim of the current study was to detect genetic biomarkers that could be implemented in national diagnostic reference laboratories to assign treatment according to biology, rather than just age, stage, and *MYCN* status in a future clinical trial. Most tumors from patients in this study harbor tSCAs.^11,39,40^ Pinto et al^11^ reported patients with tumor 11q loss had inferior EFS and OS in multivariable analyses, which we found significantly associated with inferior EFS only. Patients with tumors with only NCAs were a minority and showed the most favorable outcomes.^4,5^ The absence of SCAs could be considered as a biomarker for treatment reduction for this subgroup in future clinical trials.

Importantly, to our knowledge, this is the first study to establish oncogene amplification and p53 pathway abnormalities as independent predictors of poor survival in this group. Although *CDK4* and *MDM2* amplification present therapeutic vulnerabilities, early-phase clinical trials of MDM2 or CDK4 inhibitors have so far been disappointing with unacceptable toxicity.^41,42^ p53 pathway alterations were found to be a highly significant determinant of unfavorable EFS and OS, despite being present in only five patients after exclusion of oncogene-amplified cases, indicating that defective p53 signaling identifies an aggressive biological phenotype that is not solely a surrogate of highly amplified genomes.

Activating *ALK* mutations were detected in 15% of tumors, at a similar frequency to high-risk neuroblastoma,^34^ but consistent with previous reports were not prognostic in this group.^12,23^

However, evaluation of the effects of ALK inhibitor therapy to facilitate tumor resection could be considered in the context of a clinical trial.

The presence of TMM was found to be associated with inferior EFS, but was excluded from multivariable analyses due to small numbers. Detection of TMM in a clinical setting is challenging and unlikely to be easily implementable in routine diagnostic laboratories. However, we suggest TMM testing is undertaken prospectively as part of a future research study in these patients alongside RNAseq for cell-state signatures. Since ALT and *MYCN* amplification rarely co-occur, we expected a higher frequency of ALT-activated tumors compared with high-risk groups.^28,43-45^ Previous studies used C-circle assays for ALT assessment, while others combined this with ALT immuno-FISH and/or *TERRA* expression.^28,29,46^ Considering the prognostic implications of ALT in neuroblastoma, validation of a clinical ALT detection method is needed. *TP53* mutations have been linked to longer telomeres in different cancers,^25^ and somatic alterations in *TP53* pathway genes have also been associated with telomere dysfunction and ALT.^28^

Consistent with a previous study,^11^ we identified a predominantly adrenergic gene expression signature in tumors with RNA-seq data. However, a mesenchymal signature conferred significantly worse EFS, but was not included in multivariable analysis due to small numbers. The catalytic component of telomerase, hTERT, may mediate the switch to a mesenchymal state.^47^ Indeed, two cases with the highest *TERT* expression in our study demonstrated a mesenchymal signature.

Our study is limited by its retrospective nature and tumor availability, with some analyses undertaken on small subsets, and in around 20% of cases, analysis was undertaken in different laboratories. Given the rarity of this patient group, this was inevitable, but through international collaboration, we have assembled one of the largest cohorts of this tumor group to date.

Clinical and genetic details of an additional five patients who received upfront MAT are shown in the Data Supplement (Table S11). In this group, four patients relapsed and one died from toxicity. This contrasts with reports of better 5-year EFS with MAT in a similar group of 101 patients with neuroblastoma treated on US protocols.^11^ However, 5-year OS in the US study was 77.7 ± 4.7%, similar to the current study at 76.3% (95% CI, 68.0 to 85.5), suggesting alternative treatments instead of upfront MAT are needed for patients with unfavorable biological profiles.

In conclusion, our study identifies a subset of patients with tumors with tSCAs, oncogene amplification, or p53 pathway alterations who had inferior outcomes and may benefit from alternative treatments such as chemoimmunotherapy. Importantly, these genetic abnormalities are identifiable through routine molecular diagnostic testing including DNA arrays for CNA and next-generation sequencing for p53 pathway abnormalities. Our study provides a rationale for biological reclassification of this group of patients in a future clinical trial where patients with unfavorable biological features receive additional or alternative treatments.

## Data Availability

A data sharing statement provided by the authors is available with this article at DOI https://doi.org/10.1200/PO-26-00045. The data generated in this study are available within the article and its supplementary data files. The DNA sequencing data generated in this study are available in the European Genome Phenome Archive EGASXXXXX. The RNAseq data have been deposited in Gene Expression Omnibus accession number GSE327331. Any additional data are available upon reasonable request from the corresponding author.
